# Impact of Minimally Invasive Surgery on Anatomic Liver Segmentectomy Using the Extrahepatic Glissonean Approach

**DOI:** 10.3390/jpm14010120

**Published:** 2024-01-20

**Authors:** Yutaro Kato, Atsushi Sugioka, Masayuki Kojima, Ichiro Uyama

**Affiliations:** 1 Department of Gastroenterological Surgery, Bantane Hospital, Fujita Health University, Nagoya 454-8509, Japan; 2 Department of Advanced Robotic and Endoscopic Surgery, Fujita Health University, Toyoake 470-1192, Japan; iuyama@fujita-hu.ac.jp; 3 International Medical Center, Fujita Health University Hospital, Toyoake 470-1192, Japan; sugioka@fujita-hu.ac.jp; 4 Department of Surgery, Fujita Health University, Toyoake 470-1192, Japan; kojima@fujita-hu.ac.jp

**Keywords:** anatomic liver resection, segmentectomy, minimally invasive liver resection, laparoscopic liver resection, robotic liver resection, posterosuperior segment, repeat hepatectomy

## Abstract

Accurate minimally invasive anatomic liver (sub)segmentectomy (MIAS) is technically demanding and not yet standardized, and its surgical outcomes are undefined. To study the impact of the minimally invasive approach on perioperative outcomes of anatomic liver (sub)segmentectomy (AS), we retrospectively studied and compared perioperative outcomes of 99 open AS (OAS) and 112 MIAS (laparoscopic 77, robotic 35) cases using the extrahepatic Glissonean approach, based on the 1:1 propensity score matched analyses. After matching (71:71), MIAS was superior to OAS in terms of blood loss (*p* < 0.0001), maximum postoperative serum total bilirubin (*p* < 0.0001), C-reactive protein (*p* = 0.034) levels, R0 resection rate (*p* = 0.021), bile leak (*p* = 0.049), and length of hospital stay (*p* < 0.0001). The matched robotic and laparoscopic AS groups (30:30) had comparable outcomes in terms of operative time, blood loss, transfusion, open conversion, postoperative morbidity and mortality, R0 resection, and hospital stay, although the rate of Pringle maneuver application (*p* = 0.0002) and the postoperative aspartate aminotransferase level (*p* = 0.002) were higher in the robotic group. Comparing the matched posterosuperior (sub)segmentectomy cases or unmatched repeat hepatectomy cases between MIAS and OAS, we observed significantly less blood loss and shorter hospital stays in MIAS. Robotic AS yielded comparable outcomes with laparoscopic AS in the posterosuperior (sub)segmentectomy and repeat hepatectomy settings, despite the worse tumor and procedural backgrounds in robotic AS. In conclusion, various types of MIAS standardized by the extrahepatic Glissonean approach were feasible and safe with more favorable perioperative outcomes than those of OAS. Although robotic AS had almost comparable outcomes with laparoscopic AS, robotics may serve to decrease the surgical difficulty of MIAS in selected patients undergoing posterosuperior (sub)segmentectomy and repeat hepatectomy.

## 1. Introduction

Anatomic liver (sub)segmentectomy (AS) is a type of hepatectomy to completely resect isolated or combined liver (sub)segment(s) determined by the third (or fourth) order division portal or Glissonean pedicles (GPs) [1,2,3]. Although accurate AS is technically demanding, it is recommended as an established type of anatomic liver resection, particularly for hepatocellular carcinoma (HCC) with potentially underlying cirrhosis or for deeply located tumors, since AS is likely to attain both surgical curability and preservation of the remnant liver volume and functional reserve [4].

In contrast to open AS (OAS) [5,6], accurate minimally invasive AS (MIAS) is not technically standardized, mainly because the techniques to safely and optimally determine the anatomically isolated liver segments remain unestablished in laparoscopic [7,8,9] or robotic surgery [10,11,12]. In addition, the optimal approaches or techniques to expose the intersegmental hepatic veins are still undefined. We have previously reported our standardized techniques of MIAS using the extrahepatic Glissonean approach (EGA) and hepatic vein-root at first cranial-to-caudal parenchymal dissection, based on the anatomical background of Laennec’s capsule of the liver [9,11,12,13,14]. A few studies from other authors reported their techniques and favorable surgical outcomes of laparoscopic AS using EGA [15,16]. However, EGA to isolate the third or fourth order division portal pedicles for AS is still challenging either in OAS or MIAS, particularly by laparoscopic techniques because of the inherent motion restrictions. On the other hand, the instrument multi-articulation and tremor filtering function in the robotic platform is expected to overcome such technical difficulty. In this context, it would be valuable to study surgical outcomes of MIAS using EGA, comparing those with OAS and those between laparoscopic and robotic AS, on which previous studies are limited [11].

Our previous study focusing on AS solely for HCC patients described the technical details of our standardized AS and demonstrated the feasibility, safety, and oncologic validity of MIAS in this population [11]. In the current study, to further examine the impact of minimally invasive surgery on AS in larger cohorts, we expanded the patient population to include non-HCC diseases and more recent HCC cases. As a result, 211 cases comprising 99 OAS and 112 MIAS cases were enrolled in this study. We herein compare perioperative outcomes of AS between the surgical approaches using propensity score matching (PSM) analyses, between OAS and MIAS or between laparoscopic and robotic AS. We further explored the potential impact of MIAS in the setting of technically challenging posterosuperior (PS) (sub)segmentectomy or repeat hepatectomy, on which few previous studies had focused [17].

## 2. Materials and Methods

### 2.1. Terminology

The terminology for liver anatomy and surgical procedures was based on the Brisbane 2000 Terminology of Liver Anatomy and Resections [1] and the Tokyo 2020 updates [2]. AS was defined as resection of a liver territory that is supplied by the third (segmentectomy) or fourth (subsegmenctectomy) order division GP or by its continuing combination. The nomenclature of the subsegmental GPs was based on the classification of the portal vein branch system proposed by Takayasu et al. [18]. Regarding the terminology of subsegmental GPs except for G4a (cranial) and G4b (dorsal), the anterior, lateral, dorsal, and medial branches of the segmental GPs were termed with adding a, b, c, and d as suffixes, such as G8a, G8b, G8c, and G8d, for example. Segment (Sg)1l corresponded to the left subsegment of the caudate lobe (Spiegel lobe). The PS (sub)segments were defined as Sg1, Sg7, and Sg8 and their anatomical subsegments, and the others were defined as anterolateral (AL) segments.

### 2.2. Surgical Indications of AS

AS was selected mostly according to the Makuuchi criteria [19]. In newly developed HCC cases where the tumor was confined to a (sub)segment, AS was the first choice from the oncologic point of view, depending on the tumor size, number, and proximity to the first or second order division GPs. In recurrent HCC cases, AS was selected when the tumor was confined to a (sub)segment and deeply located or near the (sub)segmental GP. In non-HCC cases, AS was variably selected when the tumor was deeply located or close to the root of a (sub)segmental GP for securing appropriate surgical margins. Selection of OAS or MIAS was based on the tumor size, number, and location and depended on the chronological background and learning curve. MIAS was basically indicated for tumors ≤ 15-cm, which could be resected in five or fewer excision sites without requiring biliary or vascular reconstruction. In this study, we included only AS cases where we used EGA to isolate the corresponding GPs, and those using the intrahepatic or transhepatic Glissonean approach were excluded.

### 2.3. Surgical Techniques

Surgeries were performed according to the techniques for AS that we previously reported [11,14]. Briefly, regardless of the location of (sub)segments to be resected, the surgical procedures were based on the following three steps: (1) extrahepatic isolation and clamping of the target (sub)segmental GPs at the hepatic hilum, using Laennec’s capsule as the dissection layer landmark, prior to any parenchymal dissection; (2) identification of the target (sub)segments to be resected as discolored area or by using the ‘negative staining’ method; and (3) cranial-to-caudal parenchymal dissection with exposure of the landmark hepatic veins, when necessary. Liver parenchyma was dissected using Cavitron Ultrasonic Surgical Aspirator (CUSA^®^) in open and laparoscopic cases, and the clamp–crush method with the forceps instrument and/or ultrasonic shears were used in robotic cases. Pringle maneuver was restricted as much as possible in open and laparoscopic cases according to our basic policy, while in robotic cases, it was intentionally used more freely for dry magnified operative view against limited assistants’ interventions.

### 2.4. Background Data Collection

The background data were collected from the patients’ medical charts. The extracted data included age, sex, body mass index (BMI), American Society of Anesthesiology—Performance Status (ASA) score, Charlson Comorbidity Index (CCI) score [20], Indocyanine green (ICG) retention rate at 15 min (ICGR15), histologically proven cirrhosis (postoperative diagnosis), and previous hepatectomy as well as tumor diagnosis, number, size, and location.

### 2.5. Perioperative Outcomes

Intraoperative outcomes were evaluated by operative time, blood loss, transfusion of any blood products, application of Pringle maneuver, open conversion (in MIAS), and operative difficulty according to the Iwate criteria (in MIAS) [21]. Inclusion of subsegmentectomy in the procedures and additional wedge resection, which may have increased the technical complexity and operative time, were evaluated in some analyses.

Postoperative outcomes were evaluated by serum levels of maximum total bilirubin (TB) aspartate aminotransferase (AST) and C-reactive protein (CRP), R0 resection (in malignancy cases), 90-day morbidity graded according to the Clavien-Dindo (C-D) classification [22], 30-day and 90-day mortality, and the length of postoperative hospital stay (LOS).

### 2.6. Subgroup Analyses

We performed two sets of subgroup analysis. First, to study the impact of minimally invasive approach to ‘technically challenging’ PS (sub)segmentectomy, we compared perioperative outcomes of PS (sub)segmentectomy between OAS and MIAS as well as between laparoscopic and robotic AS. Second, as AS in the repeat hepatectomy setting is also a demanding but essential procedure, we addressed the potential impact of MIAS in this setting by comparing the outcomes between OAS and MIAS and between laparoscopic and robotic AS.

### 2.7. Statistical Analysis

Continuous data were expressed as a median with range for background data or a median with interquartile range (IQR) for perioperative outcomes and were compared using the Kruskal–Wallis test. Categorical data were compared using the chi-square test. In some comparative studies, 1:1 PSM was conducted. In studies comparing the entire OAS (n = 99) and MIAS (n = 112) cohorts, the following 10 variables were matched for PSM: age, sex, BMI (<25.0/≥25.0, kg/m^2^), ASA class (I or II/≥III), ICGR15 (<13.0%/≥13.0%), tumor diagnosis (HCC/non-HCC), tumor number (single/multiple), tumor size (<3.0/≥3.0, cm), tumor location (AL/PS), and previous hepatectomy. In studies comparing laparoscopic (n = 77) with robotic (n = 35) AS, age, sex, tumor diagnosis, tumor number, tumor size, and previous hepatectomy were matched. In studies comparing OAS (n = 53) with MIAS (n = 66) for PS (sub)segmentectomy, the following seven variables were matched between the groups: age, sex, ASA class, tumor diagnosis, number, size, and history of previous hepatectomy.

The PSM method was the nearest neighborhood method with a caliper width of 0.20. A standard mean deviation <0.20 was confirmed for matched variables. *p* < 0.050 was considered statistically significant. JMP^®^ software ver. 14.0 (SAS Institute, Cary, NC, USA) was used for statistical analyses.

### 2.8. Ethical Issue

The study was conducted under approval by the institutional regulation board (Fujta Health University Institutional Regulation Board, approval number: HM19-064, approval date: 26 June 2019) and in accordance with the Declaration of Helsinki (2000).

## 3. Results

A total of 211 cases (99 OAS and 112 MIAS) undergoing AS using EGA without concomitant extrahepatic procedures or additional biliary or vascular reconstructive procedures were retrospectively identified at Fujita Health University Hospital between 2010 and April 2023. The MIAS group consisted of 77 laparoscopic and 35 robotic cases. The resected (sub)segments and surgical approaches were listed in Table 1. As shown, we performed a variety of types of resections both in OAS and MIAS cases, including not only simple full isolated segmentectomies but also subsegmentectomies or combinatory (sub)segmentectomies. The cases undergoing PS (sub)segmentectomy accounted for 56.4% (n = 119) of all.

### 3.1. Period of Operation, Case Number, and Procedural Difficulty

#### 3.1.1. Annual Numbers AS Cases

**Figure 1:**
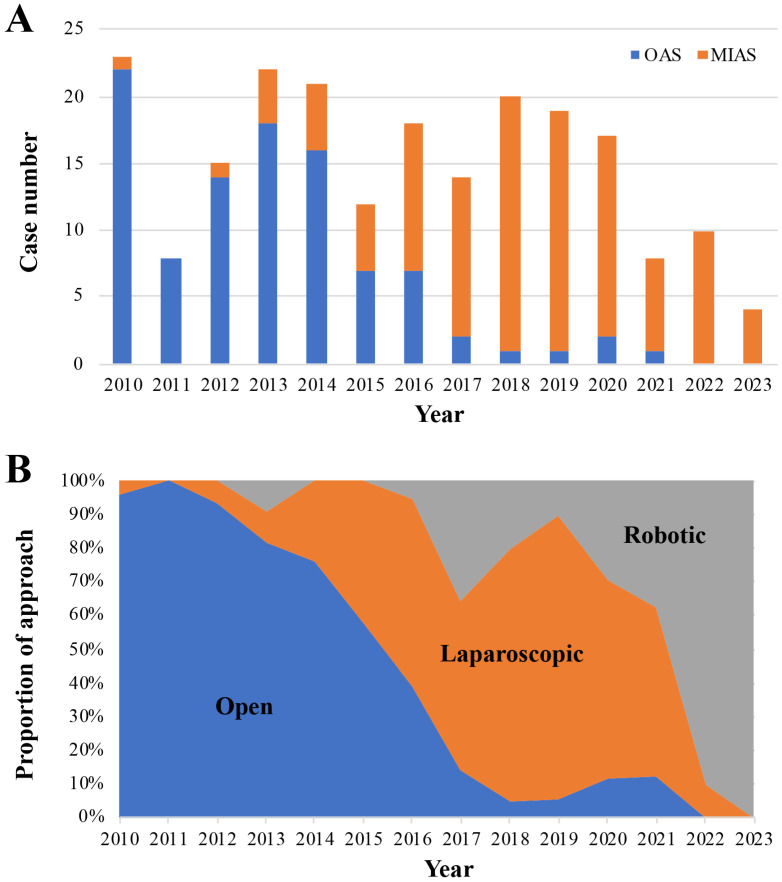
Period of operation. (**A**): Annual case numbers of OAS and MIAS from 2010 to April 2023. (**B**): Trends of the proportion of the surgical approaches in AS.

Annual case numbers of OAS and MIAS (Figure 1A) and the proportion of the surgical approach (open, laparoscopic, and robotic) to AS in each year from 2010 to 2023 (Figure 1B) were shown. The total annual case numbers of AS gradually decreased from 2021. OAS tended to be replaced by MIAS in number and the proportion of approaches year by year. Furthermore, the robotic approach tended to replace the laparoscopic approach from 2022.

#### 3.1.2. Changes in the Proportion of the MIAS Approach and Surgical Difficulty Levels

**Figure 2:**
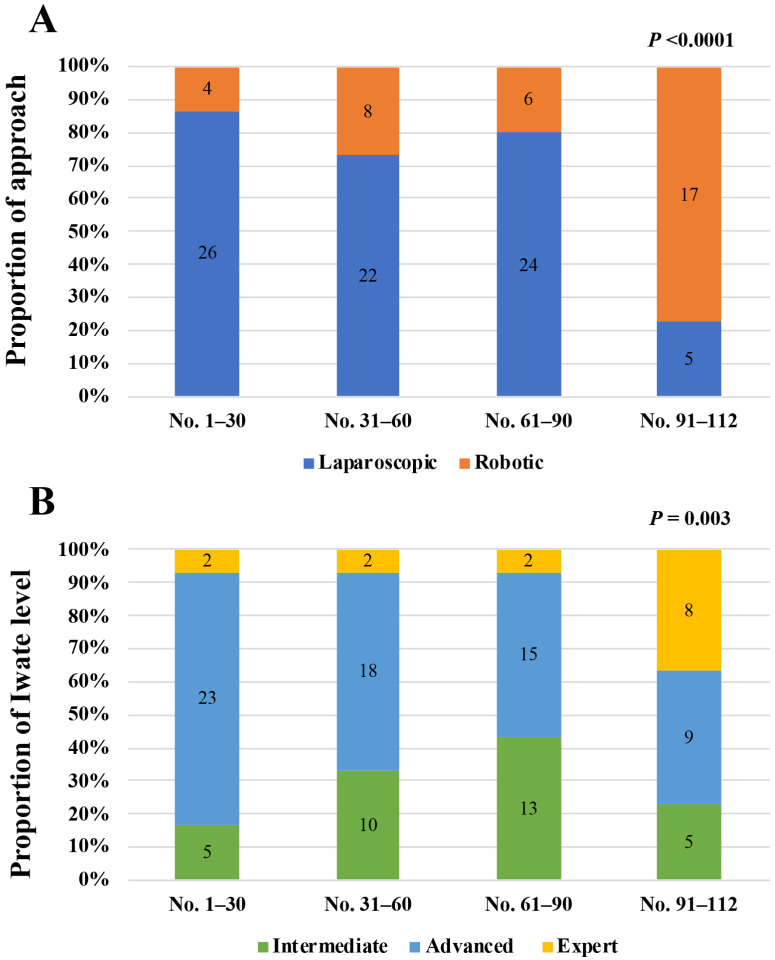
Trends of the proportion of MIAS approach (**A**) and the Iwate difficulty level (**B**) according to the accumulated MIAS case number (every 30 cases). Numbers in the bars correspond to the actual case numbers.

Figure 2A showed changes in the proportion of laparoscopic and robotic AS approaches among the four periods according to the accumulated MIAS cases by 30 cases (Case No. 1–30, No. 31–60, No. 61–90, and No. 91–112). The proportion of the robotic approach significantly increased in the most recent period (Case No. 91–112) (*p* < 0.0001). We also compared the proportion of the Iwate difficulty levels of MIAS cases among the four periods (Figure 2B). The proportion of the Iwate levels significantly changed through the four periods ((*p* = 0.003). MIAS cases in the Iwate advanced level tended to decrease as the case number increased until the period of Case No. 61–90, although the expert level cases increased in the most recent period (Case No. 91–112).

### 3.2. Perioperative Outcomes

#### 3.2.1. Comparison between OAS and MIAS

##### Background Data (Table 2)

Before PSM, compared to OAS (n = 99), MIAS (n = 112) was associated with the significantly lower ASA class (I or II class: 94.6% vs. 75.8%, *p* < 0.0001), lower CCI score (6 vs. 7 points, *p* = 0.003) and lower ICGR15 (10.5% vs. 13.7%, *p* = 0.002; ≥13.0%: 34.2% vs. 51.6%, *p* = 0.012). Further, MIAS tended to be associated with the smaller number of tumors (*p* = 0.074) and fewer cases with additional wedge resection (*p* = 0.058). After 1:1 PSM (71:71), the OAS and MIAS groups were comparable for all listed variables.

**Table 2:** Comparison of background data between OAS and MIAS cohorts.

	Before PSM			After PSM		
	OAS (n = 99)	MIAS (n = 112)	*p*	OAS (n = 71)	MIAS (n = 71)	*p*
Age, years	71 (65–75)	70 (63–74)	0.165	70 (63–75)	71 (67–76)	0.433
Sex, M/F	77/22	85/27	0.746	54/17	52/19	0.700
BMI, ≥25.0 kg/m^2^	27 (27.3)	29 (25.9)	0.821	19 (26.8)	20 (28.2)	0.851
ASA score, I or II	75 (75.8)	106 (94.6)	**<0.0001**	64 (90.1)	65 (91.5)	0.771
CCI score	7 (2–12)	6 (0–12)	**0.003**	7 (2–12)	7 (2–11)	0.788
Cirrhosis (histology)	33 (33.3)	36 (32.1)	0.854	26 (36.6)	21 (29.6)	0.373
ICGR15, %	13.7 (9.0–20.2)	10.5 (6.4–14.9)	**0.002**	12.5 (8.9–20.2)	13.0 (7.8–17.7)	0.516
≥13.0%	49 (51.6)	38 (34.2)	**0.012**	33 (46.5)	35 (49.3)	0.737
HCC	75 (75.7)	86 (76.8)	0.861	53 (74.7)	50 (70.4)	0.573
Tumor number						
Single/Multiple	66/33 (33.3)	87/25 (22.3)	0.074	48/23 (32.4)	50/21 (29.6)	0.717
Tumor size, cm	3.2 (2.2–4.8)	3.0 (2.0–4.0)	0.198	3.3 (2.4–5.0)	3.1 (2.1–4.0)	0.485
≥3.0 cm	57 (57.6)	58 (51.8)	0.399	42 (59.2)	40 (56.3)	0.734
Location, AL/PS	46/53 (53.5)	46/66 (58.9)	0.431	31/40 (56.3)	31/40 (56.3)	1.000
Including subsegmentectomy	29 (29.3)	37 (33.0)	0.558	21 (29.6)	19 (26.8)	0.709
Additional wedge resection	21 (21.2)	13 (11.6)	0.058	13 (18.3)	11 (15.5)	0.654
Previous hepatectomy	18 (18.2)	24 (21.4)	0.556	12 (16.9)	12 (16.9)	1.000

PSM: propensity score matching, OAS, and MIAS: open and minimally invasive anatomic liver (sub)segmentectomy, respectively, Continuous data: median (IQR), Categorial data: number (%), BMI: body mass index, ASA: American Society of Anesthesiology, CCI score: Charlson Comorbidity Index score; median (range), ICGR 15: indocyanine green retention rate at 15 min, HCC: hepatocellular carcinoma, AL: anterolateral, PS: posterosuperior, including subsegmentectomy; procedures including anatomic subsegmentectomy. Bold: statistically significant.

##### Perioperative Outcomes (Table 3)

Before PSM, compared to OAS, MIAS was significantly associated with longer operative time (634 vs. 554 min, *p* = 0.026), less blood loss (204 vs. 809 g, *p* < 0.0001), a lower transfusion rate (15.2% vs. 46.5%, *p* < 0.0001), a higher rate of Pringle maneuver application (26.8% vs. 7.1%, *p* = 0.0002), the lower postoperative serum maximum TB (1.5 vs. 2.2 mg/dL, *p* < 0.0001), higher maximum AST (630 vs. 409 IU/L, *p* = 0.003), lower maximum CRP (9.1 vs. 10.9 mg/dL, *p* = 0.007) levels, a higher R0 resection rate (100% vs. 94.9%, *p* = 0.015), and shorter LOS (15 vs. 26 days, *p* < 0.0001). MIAS tended to yield lower rates of 90-day overall (≥C-D I) morbidity (*p* = 0.057) and 90-day mortality (*p* = 0.064) without statistical significance.

**Table 3:** Comparison of perioperative outcomes between OAS and MIAS cohorts.

	Before PSM			After PSM		
	OAS (n = 99)	MIAS (n = 112)	*p*	OAS (n = 71)	MIAS (n = 71)	*p*
Operative time, min	554 (452–707)	634 (525–768)	**0.026**	553 (467–674)	599 (468–737)	0.266
Blood loss, g	809 (413–1413)	204 (102–492)	**<0.0001**	809 (451–1383)	215 (83–500)	**<0.0001**
Transfusion	46 (46.5)	17 (15.2)	**<0.0001**	30 (42.3)	13 (18.3)	**0.002**
Pringle maneuver	7 (7.1)	30 (26.8)	**0.0002**	3 (4.2)	18 (25.4)	**0.0004**
Open conversion	NA	1 (0.9)	NA	NA	1 (1.4)	NA
Max-TB, mg/dL	2.0 (1.4–2.9)	1.5 (1.2–1.9)	**<0.0001**	2.0 (1.4–2.8)	1.4 (1.2–1.8)	**<0.0001**
Max-AST, IU/L	409 (289–773)	630 (316–1081)	**0.003**	416 (305–723)	522 (290–1088)	0.165
Max-CRP, mg/dL	10.9 (7.6–14.4)	9.1 (6.4–12.7)	**0.007**	10.7 (7.3–13.8)	9.1 (6.5–12.4)	**0.034**
R0 resection	92 (94.9)	112 (100)	**0.015**	64 (92.8)	71 (100)	**0.021**
Morbidity ≤ 90 days						
Overall (≥C-D I)	50 (50.5)	42 (37.5)	0.057	38 (53.5)	28 (39.4)	0.093
Major (≥C-D IIIa)	16 (16.2)	13 (11.6)	0.338	12 (16.9)	6 (8.5)	0.130
Bile leak/collection	9 (9.1)	7 (6.3)	0.437	8 (11.3)	2 (2.8)	**0.049**
Mortality						
≤30 days	1 (1.0)	0 (0)	0.286	1 (1.4)	0 (0)	0.316
≤90 days	3 (3.0)	0 (0)	0.064	3 (4.2)	0 (0)	0.080
Length of hospital stay, days	26 (19–34)	15 (12–20)	**<0.0001**	24 (17–33)	16 (12–20)	**<0.0001**

Continuous data: median (IQR), Categorical data: number (%), OAS and MIAS: open and minimally invasive anatomic liver (sub)segmentectomy, NA: not applicable, Max-: postoperative maximum serum level, TB: total bilirubin, AST: aspartate aminotransferase, CRP: C-reactive protein, R0: no macroscopic residual tumors, C-D: Clavien–Dindo classification. Bold: statistically significant.

After 1:1 PSM (71:71), compared to OAS, MIAS was significantly associated with less blood loss (215 vs. 809 g, *p* < 0.0001), a lower transfusion rate (18.3% vs. 42.3%, *p* = 0.002), a higher rate of Pringle maneuver usage (25.4% vs. 4.2%, *p* = 0.0004), the lower maximum TB (1.4 vs. 2.0 mg/dL, *p* < 0.0001) and CRP (9.1 vs. 10.7 mg/dL, *p* = 0.034) levels, a higher R0 resection rate (100% vs. 92.8%, *p* = 0.021), a lower rate of bile leak (2.8% vs. 11.3%, *p* = 0.049), and shorter LOS (16 vs. 24 days, *p* < 0.0001). MIAS tended to have lower rates of 90-day overall (≥C-D I) morbidity (*p* = 0.093) and 90-day mortality (*p* = 0.080) without statistical significance.

#### 3.2.2. Comparison between Laparoscopic and Robotic AS

To study the potential impact of robotics on MIAS, we compared perioperative outcomes between laparoscopic and robotic AS cases.

##### Background Data (Table 4)

Before PSM, compared to laparoscopic AS (n = 77), robotic AS (n = 35) was associated with a significantly lower rate of cirrhosis (17.1% vs. 39.0%, *p* = 0.022), higher rates of tumor multiplicity (multiple: 34.2% vs. 16.9%, *p* = 0.040), inclusion of subsegmentectomy in the procedure (48.6% vs. 27.3%, *p* = 0.027) and repeat hepatectomy (37.1% vs. 14.3%, *p* = 0.006), and the higher number of previous hepatectomies (≥2 times: 20.0% vs. 0%, *p* < 0.0001). The robotic group tended to have the lower ASA score, smaller tumor size, and more cases undergoing additional wedge resections without statistical significance. After 1:1 PSM (30:30), all variables were comparable between the groups, except for a higher rate of inclusion of subsegmentectomy (46.7% vs. 20.0%, *p* = 0.028) and the higher number of previous hepatectomies (13.3% vs. 0%, *p* = 0.038) in robotic AS.

**Table 4:** Comparison of background data between laparoscopic and robotic AS cohorts.

	Before PSM			After PSM		
	Laparoscopic (n = 77)	Robotic (n = 35)	*p*	Laparoscopic (n = 30)	Robotic (n = 30)	*p*
Age, years	70 (62–74)	72 (64–74)	0.561	71 (66–73)	71 (63–74)	0.947
Sex, male/female	57/20	28/7	0.493	26/4	24/6	0.488
BMI, ≥25.0 kg/m^2^	21 (27.3)	8 (22.9)	0.621	7 (23.3)	7 (23.3)	1.000
ASA score, I or II	75 (97.4)	31 (88.6)	0.054	29 (96.7)	27(90.0)	0.300
CCI score	6 (0–12)	6 (3–11)	0.716	7 (2–9)	6 (3–11)	0.693
Cirrhosis (histology)	30 (39.0)	6 (17.1)	**0.022**	11 (36.7)	6 (20.0)	0.152
ICGR15, %	10.3 (6.5–14.6)	11.1 (5.9–15.8)	0.952	11.2 (7.8–14.7)	10.6 (5.9–15.8)	0.411
≥13.0%	25 (32.9)	13 (37.1)	0.661	10 (33.3)	10 (33.3)	1.000
HCC	58 (75.3)	28 (80.0)	0.587	22 (73.3)	25 (83.3)	0.347
Tumor number						
Single/Multiple	64/13 (16.9)	23/12 (34.3)	**0.040**	21/9 (30.0)	23/7 (23.3)	0.559
Tumor size, cm	3.0 (2.2–4.0)	2.5 (2.0–3.3)	0.071	3.0 (2.1–3.8)	2.7 (2.0–3.5)	0.584
≥3.0 cm	44 (57.1)	14 (40.0)	0.092	15 (50.0)	14 (46.7)	0.796
Location, AL/PS	30/47 (61.0)	16/19 (54.3)	0.501	11/19 (63.3)	15/15 (50.0)	0.297
Including subsegmentectomy	21 (27.3)	17 (48.6)	**0.027**	6 (20.0)	14 (46.7)	**0.028**
Additional wedge resection	6 (7.8)	7 (20.0)	0.062	5 (16.7)	4 (13.3)	0.718
Iwate difficulty level; Advanced or Expert	55 (71.4)	24 (68.6)	0.759	22 (73.3)	20 (66.7)	0.573
Previous hepatectomy	11 (14.3)	13 (37.1)	**0.006**	7 (23.3)	8 (26.7)	0.766
Number ≥2 times	0 (0)	7 (20.0)	**<0.0001**	0 (0)	4 (13.3)	**0.038**

PSM: propensity score matching, Continuous data: median (IQR), Categorical data: number (%), BMI: body mass index, ASA: American Society of Anesthesiology, CCI: Charlson Comorbidity Index, ICGR 15: indocyanine green retention rate at 15 min, HCC: hepatocellular carcinoma, AL: anterolateral, PS: posterosuperior. Bold: statistically significant.

##### Perioperative Outcomes (Table 5)

Before PSM, laparoscopic (n = 77) and robotic (n = 35) AS had comparable outcomes, except for a significantly higher rate of Pringle maneuver application (81.4% vs. 15.6%, *p* < 0.0001) and the higher maximum AST level (1028 vs. 512 IU/L, *p* = 0.0004) in robotic AS. Similarly, after PSM (30:30), robotic AS had a significantly higher rate of Pringle maneuver application (50.0% vs. 6.8%, *p* = 0.0002) and the higher maximum AST level (1212 vs. 475 IU/L, *p* = 0.002). The 90-day morbidity and mortality and LOS were comparable.

**Table 5:** Comparison of perioperative outcomes between laparoscopic and robotic AS cohorts.

	Before PSM			After PSM		
	Laparoscopic (n = 77)	Robotic (n = 35)	*p*	Laparoscopic (n = 30)	Robotic (n = 30)	*p*
Operative time, min	618 (502–735)	663 (545–897)	0.094	589 (503–704)	662 (544–868)	0.209
Blood loss, g	193 (96–449)	225 (105–647)	0.522	192 (108–358)	180 (72–578)	0.994
Transfusion	12 (15.9)	5 (14.3)	0.859	4 (13.3)	4 (13.3)	1.000
Pringle maneuver	12 (15.6)	18 (51.4)	**<0.0001**	2 (6.8)	15 (50.0)	**0.0002**
Open conversion	0 (0)	1 (2.9)	0.136	0 (0)	0 (0)	1.000
Max-TB, mg/dL	1.4 (1.2–1.9)	1.5 (1.4–2.0)	0.383	1.4 (1.2–1.9)	1.5 (1.2–2.0)	0.523
Max-AST, IU/L	512 (289–864)	1028 (526–2279)	**0.0004**	475 (324–855)	1212 (558–2885)	**0.002**
Max-CRP, mg/dL	8.9 (6.5–12.4)	10.1 (6.1–14.1)	0.367	9.1 (6.7–12.5)	10.3 (6.0–14.1)	0.534
R0 resection	77 (100)	35 (100)	1.000	30 (100)	30 (100)	1.000
Morbidity ≤ 90 days						
Overall (≥C-D I)	25 (32.5)	17 (48.6)	0.103	9 (30.0)	12 (40.0)	0.417
Major (≥C-D IIIa)	6 (7.8)	7 (20.0)	0.062	1 (3.3)	4(13.3)	0.161
Bile leak/collection	5 (6.5)	2 (5.7)	0.875	1 (3.3)	1 (3.3)	1.000
Mortality ≤ 90 days	0 (0)	0 (0)	1.000	0 (0)	0 (0)	1.000
Length of hospital stay, days	15 (12–19)	16 (13–21)	0.219	15 (12–20)	16 (11–21)	0.495

Continuous data: median (IQR), Categorical data: number (%), Max-: postoperative maximum serum level, TB: total bilirubin AST: aspartate aminotransferase, CRP: C-reactive protein, C-D: Clavien–Dindo classification. Bold: statistically significant.

#### 3.2.3. Subgroup Analysis

##### Anatomic PS (sub)Segmentectomy (Table 6)

Anatomic PS (sub)segmentectomy was performed in 53.5% (n = 53), 61.0% (n = 47), and 54.3% (n = 19) of open, laparoscopic, and robotic cases, respectively, and the proportion was comparable among the approaches. In comparison, before PSM between OAS (n = 53) and MIAS (n = 66), background data were comparable except for the significantly higher ASA score class (*p* = 0.0003) and CCI score (*p* = 0.022) in OAS. After PSM (40:40), all studied variables became comparable.

**Table 6:** Perioperative outcomes of posterosuperior (sub)segmentectomy.

	Before PSM			After PSM			Laparoscopic (n = 47)	Robotic (n = 19)	*p* ***
	OAS (n = 53)	MIAS (n = 66)	*p* *	OAS (n = 40)	MIAS (n = 40)	*p* **			
Age, years	73 (44–91)	71 (36–86)	0.087	72 (44–91)	71 (36–85)	0.630	71 (36–86)	72 (55–82)	0.766
Sex, male/female	38/15	48/18	0.908	29/11	28/12	0.805	34/13	14/5	0.912
ASA score, I or II	38 (71.7)	63 (95.5)	**0.0003**	37 (92.5)	37 (92.5)	1.000	46 (97.8)	17 (89.5)	0.138
CCI score	7 (2–12)	7 (2–12)	**0.022**	7 (2–12)	7 (2–12)	0.313	7 (2–12)	7 (4–10)	0.645
Cirrhosis (histology)	36 (67.9)	40 (60.6)	0.409	14 (35.0)	17 (42.5)	0.491	22 (46.8)	4 (21.1)	0.053
ICGR15 ≥13.0%	25 (49.0)	25 (37.9)	0.227	18 (47.4)	16 (40.0)	0.512	15 (31.9)	10 (52.6)	0.116
HCC	42 (79.3)	52 (78.8)	0.952	31 (77.5)	32 (80.0)	0.785	38 (80.9)	14 (73.7)	0.519
Tumor number, Multiple	17 (32.1)	17 (25.8)	0.448	12 (30.0)	9 (22.5)	0.446	7 (14.9)	10 (52.6)	**0.002**
Tumor size, ≥3.0 cm	32 (60.4)	37 (56.1)	0.635	24 (60.0)	24 (60.0)	1.000	29 (61.7)	8 (42.1)	0.146
Including subsegmentectomy	21 (39.6)	25 (37.9)	0.846	15 (37.5)	18 (45.0)	0.496	15 (31.9)	10 (52.6)	0.116
Additional wedge resection	10 (18.8)	8 (12.1)	0.307	8 (20.0)	5 (12.5)	0.363	2 (4.3)	6 (31.6)	**0.002**
Repeat hepatectomy	8 (15.1)	13 (19.7)	0.513	7 (17.5)	6 (15.0)	0.762	6 (12.8)	7 (36.8)	**0.026**
Operative time, min	622 (530–786)	706 (570–884)	**0.024**	632 (550–792)	697 (568–852)	0.260	681 (567–829)	858 (582–1055)	0.064
Blood loss, g	1053 (561–1563)	353 (162–756)	**<0.0001**	1075 (606–1611)	271 (154–683)	**<0.0001**	300 (158–655)	631 (162–1116)	0.088
Open conversion	NA	1 (1.5)	NA	NA	1 (2.5)	NA	0 (0)	1 (5.3)	0.113
R0 resection	49 (92.5)	66 (100)	**0.023**	37 (92.5)	40 (100)	0.078	47 (100)	19 (100)	1.000
Morbidity ≤ 90 days									
Overall (≥ C-D I)	32 (60.4)	28 (42.4)	0.052	25 (62.5)	25 (62.5)	**0.025**	19 (40.4)	9 (47.4)	0.605
Major (≥ C-D IIIa)	11 (20.8)	10 (15.2)	0.426	6 (15.0)	6 (15.0)	1.000	5 (10.6)	5 (26.3)	0.108
Bile leak/Collection	5 (9.4)	4 (6.1)	0.489	3 (7.5)	3 (7.5)	1.000	3 (6.4)	1 (5.3)	0.863
Mortality ≤ 90 days	3 (5.7)	0 (0)	0.050	2 (5.0)	0 (0)	0.152	0 (0)	0 (0)	1.000
Length of hospital stay, days	29 (22–41)	16 (13–21)	**<0.0001**	29 (21–36)	15 (11–18)	**<0.0001**	16 (12–20)	16 (14–24)	0.580

Continuous data: median (range or IQR), Categorial data: number (%), OAS and MIAS: open and minimally invasive anatomic (sub)segmentectomy, ASA: American Society of Anesthesiology score, CCI: Carlson Comorbidity Index, ICGR15: indocyanine green retention rate at 15 min, HCC: hepatocellular carcinoma, Including subsegmentectomy: procedures including anatomic subsegmentectomy, NA: not applicable, C-D: Clavien–Dindo grade, *p* *: OAS vs. MIAS (before PSM), *p* **: OAS vs. MIAS (after PSM), *p* ***: Laparoscopic vs. Robotic (non-PSM). Bold: statistically significant.

Regarding the postoperative outcomes, before PSM, MIAS was significantly associated with longer operative time (706 vs. 622 min, *p* = 0.024), less blood loss (353 vs. 1053 g, *p* < 0.0001), a higher R0 resection rate (100% vs. 92.5%, *p* = 0.023), and shorter LOS (16 vs. 29 days, *p* < 0.0001). After PSM, operative time became comparable, and MIAS had significantly less blood loss (271 vs. 1075 g, *p* < 0.0001), a lower rate of overall (≥CD-I) morbidity (37.5% vs. 62.5%, *p* = 0.025), and shorter LOS (15 vs. 29 days, *p* < 0.0001).

In non-PSM comparison of background data between laparoscopic (n = 47) and robotic (n = 19) AS, the latter was associated with a significantly larger tumor number (multiple: 52.6% vs. 14.9%, *p* = 0.002), more cases with additional wedge resections (31.6% vs. 4.3%, *p* = 0.002), and a higher rate of repeat hepatectomy setting (36.8% vs. 12.8%, *p* = 0.026). Perioperative outcomes were comparable between the groups.

##### AS in the Repeat Hepatectomy Setting (Table 7)

AS was performed for post-hepatectomy recurrent liver tumors in 42 cases (19.9%), including 18 OAS (18.2%) and 24 MIAS (21.4%) cases. Such repeat hepatectomy cases accounted for 18.2% (n = 18), 14.3% (n = 11), and 37.1% (n = 13) of all open, laparoscopic, and robotic AS cases, respectively, and a significantly higher number of cases were performed by robotic surgery (*p* = 0.016).

**Table 7:** Perioperative outcomes of AS in the repeat hepatectomy setting.

	OAS (n = 18)	MIAS (n = 24)	*p* *	Laparoscopic (n = 11)	Robotic (n = 13)	*p* **
Age, years	74 (68–75)	70 (68–74)	0.646	70 (67–71)	72 (69–77)	0.416
Sex, M/F	15/3	19/5	0.734	10/1	9/4	0.193
CCI score	7 (4–12)	6 (2–10)	0.391	6 (2–10)	6 (4–9)	0.976
Cirrhosis (histology)	6 (33.3)	10 (41.7)	0.582	6 (54.6)	4 (30.8)	0.239
ICGR15 ≥ 13.0%	7 (41.2)	9 (39.1)	0.896	2 (20.0)	7 (53.9)	0.099
HCC	14 (77.8)	19 (79.2)	0.914	8 (72.7)	11 (84.6)	0.475
Multiple tumors	5 (27.8)	5 (20.8)	0.601	0 (0)	5 (38.5)	**0.021**
Tumor size, ≥3.0 cm	9 (50.0)	5 (20.8)	**0.047**	3 (27.3)	2 (15.4)	0.475
Previous Hx ≥2 times	0 (0)	7 (29.2)	**0.012**	0 (0)	7 (53.9)	**0.004**
Previous open Hx	15 (83.3)	12 (50.0)	**0.026**	3 (27.3)	9 (69.2)	**0.041**
Iwate difficulty level, Advanced or Expert	NA	14 (58.3)	NA	6 (54.6)	8 (61.5)	0.729
Operative time, min	533 (439–780)	658 (547–750)	0.112	654 (546–751)	661 (534–829)	0.931
Blood loss, g	514 (323–1579)	149 (87–945)	**0.004**	153 (114–994)	138 (68–957)	0.839
Open conversion	NA	1 (4.2)	NA	0 (0)	1 (7.7)	0.347
R0 resection	17 (94.4)	24 (100)	0.243	11 (100)	13 (100)	1.000
Morbidity ≤ 90 days						
Overall (≥C-D I)	9 (50.0)	13 (54.2)	0.789	4 (36.3)	9 (69.2)	0.107
Major (≤C-D IIIa)	3 (16.7)	5 (20.8)	0.734	1 (9.1)	4 (30.8)	0.193
Bile leak/Collection	2 (11.1)	3 (12.5)	0.891	1 (9.1)	2 (15.4)	0.643
Mortality ≤ 90 days	0 (0)	0 (0)	1.000	0 (0)	0 (0)	1.000
LOS, days	24 (20–36)	17 (13–21)	**0.005**	15 (11–18)	19 (16–24)	0.087

Continuous data: median (range or IQR), Categorial data: number (%), OAS and MIAS: open and minimally invasive anatomic (sub)segmentectomy, *p* *: OAS vs. MIAS, *p* **: Laparoscopic vs. Robotic, CCI: Charlson Comorbidity Index, ICGR15: indocyanine green retention rate at 15 min, HCC: hepatocellular carcinoma, Hx: hepatectomy, C-D: Clavien–Dindo grade, LOS: length of postoperative hospital stay. Bold: statistically significant.

Compared to OAS, MIAS was associated with the significantly smaller tumor size (≥3.0 cm: 20.8% vs. 50.0%, *p* = 0.047), the larger number of previous hepatectomies (≥2 times: 29.2% vs. 0%, *p* = 0.012), and fewer cases with prior open hepatectomy (50.0% vs. 83.3%, *p* = 0.026) (Table 7). Regarding the perioperative outcomes, MIAS had significantly less blood loss (149 vs. 514 g, *p* = 0.004) and shorter LOS (17 vs. 24 days, *p* = 0.005) than OAS.

Compared to laparoscopic AS, robotic AS was associated with the significantly higher tumor number (multiple: 38.5% vs. 0%, *p* = 0.021), higher number of previous hepatectomies (≥2 times: 53.9% vs. 0%, *p* = 0.004), and more cases undergoing prior open hepatectomy (69.2% vs. 27.3%, *p* = 0.041). Perioperative outcomes were comparable between the groups.

## 4. Discussion

In this study, we examined perioperative outcomes of 211 patients who underwent OAS or MIAS using EGA, encompassing those with HCC, colorectal metastasis, and other types of liver tumors. Compared to our previous study on AS for HCC only [11], the current study enrolled larger number of patients with more various indications, levels of liver functional reserve, and types of resection. The resultant increase in the sample size may have contributed to higher statistical reliability and enabled us to perform two sets of subgroup analysis on anatomic PS (sub)segmentectomy and AS in the repeat hepatectomy setting, on which the impact of minimally invasive or robotic approach has been poorly evaluated previously. Furthermore, the surgical team was the same throughout the study period and performance of surgery using the consistent EGA-based AS techniques by the expert surgeons (Y.K., A.S., I.U.) or by the non-expert surgeons under strict intraoperative instruction by the above expert surgeons could have reduced technical biases. In such study settings, a variety of surgical procedures were safely performed both in OAS and MIAS, suggesting the feasibility and safety of EGA to AS, regardless of the type of resection and surgical approach.

The characteristic trends showing the replacement of OAS by MIAS from 2016 (Figure 1A) and that of laparoscopic AS by robotic AS from 2022 (Figure 1B) can be explained by the start of national insurance cover of the laparoscopic and robotic AS approach in these years, respectively, in addition to the learning curves of the relatively newer surgical approaches. Furthermore, an increase in the proportion of the robotic approach coincided with that in the proportion of MIAS cases with the higher Iwate difficulty levels in the most recent period after we experienced 90 cases (Figure 2). This may be attributed to a learning curve of the robotic approach and our tendency to select this approach in technically difficult AS cases.

The PSM-based analyses on OAS and MIAS showed that MIAS was superior to OAS in terms of blood loss, transfusion, postoperative TB and CRP levels, R0 resection, bile leak, and LOS. These results are partly in line with those of previous studies in several types of anatomic resection [9,11,12,14,23,24], though the significantly lower CRP level, a higher R0 resection rate, and a lower rate of bile leak in MIAS than in OAS seem to be novel findings. The higher postoperative AST level in MIAS may be related to the higher rate of Pringle maneuver application in robotic AS, where it was used freely in this series.

Subgroup PSM-based analyses on PS (sub)segmentectomy showed that, compared to OAS, MIAS was associated with significantly less blood loss, a lower rate of transfusion, a higher R0 resection rate, and shorter LOS, though with longer operative time. These results partly agree with those of previous studies on parenchyma-sparing PS resection [9,25], though few studies selectively focused on minimally invasive posterosuperior AS [10,26]. Between laparoscopic and robotic AS, perioperative outcomes were comparable, despite the more challenging background in the latter, including a higher tumor number, more cases undergoing concomitant wedge resections, and a higher rate of repeat hepatectomy setting. These results may collectively suggest the potential advantages of MIAS over OAS and those of robotic over laparoscopic AS in selected patients undergoing anatomic PS (sub)segmentectomy.

On the other hand, in the PSM-based comparison between laparoscopic and robotic AS, the outcomes were comparable except for the higher AST level and more frequent application of Pringle maneuver in robotic AS. Of note, compared to the laparoscopic AS group, the robotic group had more technically demanding cases including EGA-based subsegmentectomy and those undergoing multiple previous hepatectomies. The mostly comparable perioperative outcomes, even upon worse backgrounds in robotic AS, may indirectly suggest potential benefits of robotics for AS in selected patients. Recent comparative studies on minimally invasive anatomic resection showed better perioperative outcomes in robotic than in laparoscopic surgery including less blood loss, less postoperative morbidity, and shorter LOS [27,28,29,30], although none of these studies included isolated or combined AS cases. Only one study including MIAS by Morimoto et al. reported less blood loss in the robotic than in the laparoscopic group [16]. Larger studies selectively focusing on AS are warranted to study the impact of robotics on this type of anatomically complex procedure.

In the repeat hepatectomy setting, MIAS is technically demanding and time-consuming and appears to be less frequently performed than wedge resection, due to its surgical difficulty and risk of unexpected impairment of remnant hepatic functional reserve. Such limitations of using MIAS for redo hepatectomy may attribute to the variable need of extensive adhesiotomy, anatomical deviation of the vital structures, and potential difficulty in hepatic inflow control including Pringle maneuver as well as the inherent motion restrictions and limited intraabdominal space for complex procedures. In this AS series, the robotic approach was more frequently used in repeat hepatectomy cases than the open or laparoscopic approach. Additionally, robotic AS was more frequently applied in cases undergoing multiple previous hepatectomies or prior open hepatectomies than laparoscopic AS. Such aggressive application of robotic surgery in redo AS cases derived from our preference and belief in the potential advantages of robotics for a minimally invasive approach in such demanding procedure. However, these circumstances produce background biases in comparative analyses. Nonetheless, despite these background handicaps in MIAS or robotic AS, MIAS had less blood loss and shorter LOS than OAS, and robotic AS had comparable outcomes with laparoscopic AS. Such satisfactory outcomes of MIAS or robotic AS may suggest their potential advantages for AS in the repeat hepatectomy setting. No previous studies have focused on MIAS including robotic AS for redo hepatectomy, and thus, although based on the small sample size, this study seems to add valuable results to the literature.

In view of more favorable perioperative outcomes in MIAS than those in OAS, the minimally invasive approach is strongly recommended for AS at least in the expert hands using EGA, particularly in cases of anatomic PS (sub)segmentectomy and repeat hepatectomy. Furthermore, robotic surgery is potentially a more reliable option for MIAS than laparoscopic surgery in these demanding cases. However, the sample size was small and larger studies are necessary to further address the potential advantages of MIAS and robotic AS.

Although in the future, MIAS would replace OAS for the majority of patients who need AS, there remains an important issue of the training of young surgeons. Previous studies on the surgical training for minimally invasive liver resection have mainly focused on the learning curves to attain procedural safety [31,32]. However, very few studies have addressed the evidence-based, seamless training paths from open to minimally invasive liver resection. A critical problem is how young liver surgeons should be trained to prepare for open conversion during MIAS, where the patient’s life can be threatened by massive bleeding or vital organ injury. With the dissemination of minimally invasive surgery, young surgeons will have less and less experience with open liver resection. On the other hand, liver cases out of the scope of minimally invasive surgery still do and will exist. Therefore, in our opinion, young surgeons should be trained on a fixed number open (relatively difficult) liver resection cases before starting minimally invasive complex resection such as MIAS.

There are several limitations in this study. First, the retrospective and observational nature of this study precludes definite conclusions, though PSM was conducted to reduce biases. Second, this is a single-center study with a small sample size. Third, there are chronological biases which affect selection of the surgical approach and learning curve.

In conclusion, MIAS using EGA is feasible and safe and would contribute to better perioperative outcomes than OAS. Robotic AS was comparable to laparoscopic AS in terms of perioperative outcomes as a whole but may potentially serve to decrease the surgical difficulty of PS (sub)segmentectomy or AS for repeat hepatectomy for recurrent tumors in selected patients.

## Figures and Tables

**Table 1:** Resected liver (sub)segments.

	OAS (n = 99)	MIAS (n = 112)		Total
		Laparoscopic (n = 77)	Robotic (n = 35)	
*Resected (sub)segments, n*				
**Sg1**	2	4	4	10
Sg1l	3	3	2	8
**Sg2**	3	4	3	10
**Sg3**	5	4	2	11
Sg3a	1	0	0	1
Sg3b	0	1	0	1
**Sg4**	3	1	0	4
Sg4b	1	1	1	3
Sg4b+8a	1	0	0	1
Sg4b+5	1	1	0	2
Sg4b+5+6a	2	0	0	2
**Sg5**	13	5	2	20
Sg5a	1	1	0	2
Sg5ab	1	0	0	1
Sg5+6a	1	1	0	2
Sg5+8b	1	0	0	1
Sg5+6	5	3	1	9
Sg5+6+8c	1	0	0	1
Sg5+Sg2	1	0	0	1
**Sg6**	8	7	2	17
Sg6a	0	1	2	3
Sg6ab	0	0	1	1
Sg6bc	0	0	1	1
Sg6+5c	0	0	1	1
**Sg7**	12	8	2	22
Sg7b	0	1	0	1
Sg7bc	0	0	1	1
Sg7bc+6c	0	1	0	1
Sg7+6c	2	0	0	2
Sg7+6bc+8c	1	0	0	1
Sg7+8	2	0	0	2
Sg7+Sg2	1	0	0	1
**Sg8**	15	20	3	38
Sg8a	3	1	1	5
Sg8b	0	1	1	2
Sg8c	4	3	2	9
Sg8ab	1	1	0	2
Sg8bc	0	1	0	1
Sg8abc	1	0	0	1
Sg8acd	0	0	1	1
Sg8bcd	1	0	0	1
Sg8a+5a	0	1	0	1
Sg8b+5+6a	0	1	0	1
Sg8c+5bc	0	1	0	1
Sg8c+5c+6c	0	0	1	1
Sg8+1r	1	0	0	1
Sg8+5b	1	0	0	1
Sg8ab+Sg1l	0	0	1	1
*Classification, n (%)*				
Anterolateral	46 (46.5)	30 (39.0)	16 (45.7)	92 (43.6)
Posterosuperior	53 (53.5)	47 (61.0)	19 (54.3)	119 (56.4)

Bold: full isolated liver segments.

## Data Availability

The datasets are not available for public access due to patient privacy concerns but are available from the corresponding author on reasonable request.
